# Early Improvement of Acute Respiratory Distress Syndrome in Patients With COVID-19 in the Intensive Care Unit: Retrospective Analysis

**DOI:** 10.2196/24843

**Published:** 2021-03-09

**Authors:** Zhu Zhan, Xin Yang, Hu Du, Chuanlai Zhang, Yuyan Song, Xiaoyun Ran, An Zhang, Mei Yang

**Affiliations:** 1 Department of Intensive Care Unit The Second Affiliated Hospital of Chongqing Medical University Chongqing China; 2 Chongqing Key Laboratory of Ultrasound Molecular Imaging The Second Affiliated Hospital of Chongqing Medical University Chongqing China; 3 Department of Respiratory and Geriatrics Chongqing Public Health Medical Center Chongqing China; 4 Department of Intensive Care Unit Chongqing Public Health Medical Center Chongqing China; 5 Department of Intensive Care Unit Chongqing Sixth People's Hospital Chongqing China

**Keywords:** acute respiratory distress syndrome, ARDS, Chongqing, COVID-19, critically ill, intensive care unit, outcome, characteristic, mortality, epidemiology, improvement

## Abstract

**Background:**

Since the start of the COVID-19 pandemic, there have been over 2 million deaths globally. Acute respiratory distress syndrome (ARDS) may be the main cause of death.

**Objective:**

This study aimed to describe the clinical features, outcomes, and ARDS characteristics of patients with COVID-19 admitted to the intensive care unit (ICU) in Chongqing, China.

**Methods:**

The epidemiology of COVID-19 from January 21, 2020, to March 15, 2020, in Chongqing, China, was analyzed retrospectively, and 75 ICU patients from two hospitals were included in this study. On day 1, 56 patients with ARDS were selected for subgroup analysis, and a modified Poisson regression was performed to identify predictors for the early improvement of ARDS (eiARDS).

**Results:**

Chongqing reported a 5.3% case fatality rate for the 75 ICU patients. The median age of these patients was 57 (IQR 25-75) years, and no bias was present in the sex ratio. A total of 93% (n=70) of patients developed ARDS during ICU stay, and more than half had moderate ARDS. However, most patients (n=41, 55%) underwent high-flow nasal cannula oxygen therapy, but not mechanical ventilation. Nearly one-third of patients with ARDS improved (arterial blood oxygen partial pressure/oxygen concentration >300 mm Hg) in 1 week, which was defined as eiARDS. Patients with eiARDS had a higher survival rate and a shorter length of ICU stay than those without eiARDS. Age (<55 years) was the only variable independently associated with eiARDS, with a risk ratio of 2.67 (95% CI 1.17-6.08).

**Conclusions:**

A new subphenotype of ARDS—eiARDS—in patients with COVID-19 was identified. As clinical outcomes differ, the stratified management of patients based on eiARDS or age is highly recommended.

## Introduction

### Background

In December 2019, Wuhan, Hubei Province, China, reported a cluster of pneumonia cases of unknown cause, later identified as COVID-19 [1]. This contagious disease is caused by SARS-CoV-2. COVID-19 was declared a pandemic by the World Health Organization (WHO) on March 11, 2020 [2]. As of January 17, 2021, 1 year has passed since the pandemic began, with more than 93 million cases and 2 million deaths reported worldwide [3].

The leading cause of COVID-19–related death may be severe respiratory failure caused by acute respiratory distress syndrome (ARDS) [4]. According to some autopsy results, the lesions primarily appear in the lungs, characterized by diffuse alveolar damage. Other organs, such as heart tissue, have no obvious histological changes [5-7]. Previous studies reported that 48.6% of patients with COVID-19 had ARDS, of whom 29% died, and the mortality rate increased with the severity of ARDS [8,9]. Therefore, the characteristics of ARDS in patients with COVID-19 need to be fully understood.

This study aimed to describe the epidemiology, clinical features, laboratory findings, treatments, and outcomes of intensive care unit (ICU) patients with COVID-19 in Chongqing, China, which neighbors Hubei Province. In addition, a subphenotype of ARDS—early improvement of ARDS (eiARDS)—which occurred in about one-third of patients, was identified. eiARDS predicted a favorable clinical outcome.

## Methods

### Study Design and Participants

This retrospective cohort study included two cohorts of ICU patients from the Chongqing Public Health Medical Center and the Chongqing Three Gorges Central Hospital (Chongqing, China), both of which were designated hospitals for the treatment of patients with COVID-19 in Chongqing. Patients admitted to the ICU between January 21, 2020 (the date the first patient was admitted), and March 15, 2020 (the date the last patient was discharged during the first wave), were enrolled in this study.

Patients with COVID-19 were confirmed by a positive real-time reverse transcription–polymerase chain reaction (RT-PCR) assay using nasal swab specimens per WHO guidance [10]. The severity of COVID-19 was judged according to the Fifth Revised Trial Version of the Novel Coronavirus Pneumonia Diagnosis and Treatment Guidance of China [11]. Patients meeting any of the following criteria were defined as having a severe course of disease: (1) respiratory distress with a respiratory rate of more than 30 breaths per minute, (2) oxygen saturation ≤93% in the resting state, and (3) arterial blood oxygen partial pressure (PaO_2_)/oxygen concentration (FiO_2_) ≤300 mm Hg. Patients meeting one of the following criteria were defined as critically ill: (1) mechanical ventilation needed as a result of respiratory failure, (2) shock, and (3) intensive care needed owing to the failure of other organs.

### Data Collection

The two designated hospitals were Grade A hospitals in China, and all case data were retrieved from their electronic case system. Epidemiological and demographic data, symptoms, underlying diseases, comorbidities, treatments, clinical course, and outcome data of the patients were recorded in a spreadsheet. Vital signs, arterial blood gas analysis, laboratory data, acute physiology and chronic health evaluation II, and the sequential organ failure assessment score were recorded on specified dates (day 0: admission to hospital; day 1: admission to ICU; day 3, day 7, and day 14) for each patient. If any question emerged regarding the case data, clarification was sought from the treating team physician. As data collection was completed, another doctor was responsible for checking and integrating the data. The proportion of pneumonia volume was calculated by the Pulmonary Infection–Assisted Diagnosis System (V1.7.1) based on computed tomography (CT) imaging.

### Definition

ARDS was diagnosed according to the Berlin definition [12]. Liver injury was diagnosed according to the following criteria: alanine aminotransferase >3 upper limit of normal (ULN), aspartate aminotransferase >3 ULN, or total bilirubin >2 ULN, regardless of a chronic liver disease diagnosis [13]. Acute kidney injury was diagnosed on the basis of serum creatinine [14]. Cardiac injury was diagnosed if the serum concentration of hypersensitive cardiac troponin T was greater than the upper limit of the reference range (>14 pg/mL). Cessation of viral shedding was defined as two consecutive negative nasal swab PCR tests (with an interval of least 24 hours).

### Statistical Analysis

SPSS 26.0 (IBM Corp) was used for statistical analysis. Normally distributed continuous variables were presented as mean (SD), and the independent Student *t* test was used for comparison between two groups. The continuous variables that did not meet the criteria for normal distribution were presented as the median (IQR), and the Mann-Whitney *U* test was used to compare differences between two groups. Categorical variables were summarized using frequencies and percentages, and the chi-square test or the Fisher exact test was used to compare two or more groups. A modified Poisson regression analysis (sandwich variance estimator) was performed to identify the predictors of eiARDS; variables with *P*<.05 in the univariate analysis were entered into the multivariate Poisson regression analysis. All tests were two sided, and *P*<.05 was considered statistically significant.

### Ethics Approval and Consent to Participate

The study was approved by the Research Ethics Committee of the Second Affiliated Hospital of Chongqing Medical University, the Chongqing Public Health Medical Center, and the Chongqing Three Gorges Central Hospital. Written informed consent was waived by the ethics committees of the designated hospitals for emerging infectious diseases.

### Availability of Data and Materials

Original data can be requested from the corresponding author.

## Results

### Clinical Characteristics of ICU Patients in Chongqing, China

From January 21 to March 15, 2020, Chongqing reported 576 new cases of COVID-19 and 6 deaths. In this study, 75 ICU patients from two hospitals were recruited, including 48 severe patients, 27 critically ill patients, and 4 deceased patients.

A comparison of clinical characteristics between the two groups is shown in Table 1. The median age of the 75 patients was 57 years, and no bias was found in the sex ratio. Smoking was more prevalent among critically ill patients than in severe patients. The most frequent chronic medical illnesses were diabetes and hypertension. The most common symptoms were cough, fever, and dyspnea. In addition, 2 patients experienced an asymptomatic period prior to hospitalization and were dyspneic without fever during their hospital stay.

ARDS developed in most patients (n=70, 93%), and more than half (n=38, 51%) had moderate ARDS (Table 1). However, most patients (n=41, 55%) were supported with high-flow nasal cannula (HFNC) oxygen therapy (21 patients also received ventilation during their stay in the ICU). Only 26 (35%) patients received noninvasive ventilation (7 patients also received invasive ventilation during their stay in the ICU), and 7 (9%) patients received invasive ventilation. Other supportive treatments included prone-position ventilation in 7 patients, extracorporeal membrane oxygenation in 3 patients, renal replacement therapy in 3 patients, and vasoconstrictive agents in 7 patients. Although bacterial pneumonia was identified by microbiological culture of sputum or alveolar lavage fluid in only 4 patients, antibacterial agents were administered to 62 patients and antifungal agents to 12 patients. The liver was the most commonly injured extrapulmonary organ, followed by the heart and kidneys. Lymphopenia was a very noteworthy feature in these patients, with a lower incidence of leukopenia and thrombocytopenia. Antiviral agents were used in all patients; the combination of an Aluvia (lopinavir and ritonavir) tablet and interferon-alpha was the most commonly used antiviral formula (n=69, 92%), and 4 patients (5.3%) also used ribavirin. Glucocorticoids and intravenous immunoglobulin were more commonly used in critically ill patients due to anti-inflammation properties and ability to neutralize SARS-CoV-2, respectively. Thymopeptide (thymosin alpha 1 or thymopentin) was used in most patients (n=63, 84%) to improve antiviral immunity. Traditional Chinese medicine, which is made by decoction of more than a dozen kinds of herbs, was used in 87% (n=65) of patients owing to potential antiviral and anti-inflammation properties.

**Table 1:** Clinical characteristics of severe or critically ill patients with COVID-19 admitted to the intensive care unit in Chongqing, China (from January 21 to March 15, 2020).

Characteristic			Total (N=75)	Severe (n=48)	Critically ill (n=27)^a^	*P* value
Age (years), median (IQR)			57 (25-75)	56 (47-70)	63 (51-69)	.57
**Sex**						.64
	Female		36 (48)	24 (50)	12 (44)	
	Male		39 (52)	24 (50)	15 (56)	
Smoking			9 (12)	1 (2)	8 (30)	.002
**Exposure**						.052
	Recent travel to Hubei Province		16 (21)	13 (27)	3 (11)	
	Contact with patients from Hubei Province		24 (32)	18 (38)	6 (22)	
	Contact with confirmed patients		17 (23)	7 (15)	10 (37)	
	No definite epidemiological link		18 (24)	10 (21)	8 (30)	
**Chronic medical illness**						
	Hypertension		14 (19)	8 (17)	6 (22)	.55
	Diabetes		20 (27)	12 (25)	8 (30)	.66
	Chronic cardiac disease		7 (9)	6 (13)	1 (4)	.40
	Chronic obstructive pulmonary disease		4 (5)	4 (8)	0 (0)	.31
	Malignancy		1 (1)	1 (2)	0 (0)	>.99
**Symptoms**						
	Fever		51 (68)	28 (58)	23 (85)	.02
	Cough		62 (83)	40 (83)	22 (81)	>.99
	Expectoration		29 (39)	21 (44)	8 (30)	.23
	Dyspnea		43 (57)	23 (48)	20 (74)	.03
	Myalgia		20 (27)	12 (25)	8 (30)	.66
	Headache		9 (12)	7 (15)	2 (7)	.58
	Diarrhea		7 (9)	4 (8)	3 (11)	>.99
**Comorbidities**						
	**Acute respiratory distress syndrome^b^**		70 (93)	43 (90)	27 (100)	.01
		None	5 (7)	5 (10)	0 (0)	
		Mild	10 (13)	9 (19)	1 (4)	
		Moderate	38 (51)	25 (52)	13 (48)	
		Severe	22 (29)	9 (19)	13 (48)	
	Pneumothorax		1 (1)	0 (0)	1 (4)	.77
	Bacterial pneumonia^c^		4 (5)	3 (6)	1 (4)	>.99
	Cardiac injury		14 (19)	7 (15)	7 (26)	.23
	Liver injury		19 (25)	10 (21)	9 (33)	.23
	Kidney injury		8 (11)	5 (10)	3 (11)	>.99
	Shock		7 (9)	0 (0)	7 (26)	.001
	Leukopenia		19 (25)	11 (23)	8 (30)	.52
	Lymphopenia		71 (95)	46 (96)	25 (93)	.95
	Thrombocytopenia		20 (27)	9 (19)	11 (41)	.04
**Treatment**						
	High-flow nasal cannula		41 (55)	20 (42)	21 (78)	.003
	**Mechanical ventilation**					
		Noninvasive	26 (35)	0 (0)	26 (96)	<.001
		Invasive	7 (9)	0 (0)	7 (26)	.001
	Prone position ventilation		7 (9)	0 (0)	7 (26)	.001
	Extracorporeal membrane oxygenation		3 (4)	0 (0)	3 (11)	.08
	Renal replacement therapy		3 (4)	0 (0)	3 (11)	.08
	Vasoconstrictive agents		7 (9)	0 (0)	7 (26)	.001
	Antiviral agents		75 (100)	48 (100)	27 (100)	—^d^
	Antibacterial agents		62 (83)	37 (77)	25 (93)	.17
	Antifungal		12 (16)	4 (8)	8 (30)	.04
	Glucocorticoids		46 (61)	22 (46)	24 (89)	<.001
	Immunoglobulin		30 (40)	13 (27)	17 (63)	.002
	Thymopeptides		63 (84)	37 (77)	26 (96)	.06
	Traditional Chinese medicine		65 (87)	41 (85)	24 (89)	.94

^a^Four patients who died in the ICU were included.

^b^ARDS stages were defined by the worst PaO_2_/FiO_2_ value.

^c^Bacterial pneumonia was confirmed by sputum or alveolar lavage fluid culture.

^d^Not applicable.

### Clinical Course and Outcomes

The clinical course and outcomes of patients with COVID-19 in Chongqing are shown in Table 2. Chongqing reported 6 deaths from COVID-19 up to March 15, 2020, with a mortality rate of 1.04% in all 576 patients. As 2 patients died in the emergency department, only 4 deceased patients with clinical data were included in this study, with a 28-day case fatality rate of 5.3% and a 28-day mechanical ventilation dependency of 1.3% among ICU patients. The duration from any initial symptom to diagnosis confirmed by PCR test was 5 days, to ARDS was 7 days, to ICU admission was 8 days, and to death was 16 days. The length of ICU stay was 13 days and hospital stay was 22 days.

**Table 2:** Clinical course and outcomes of patients with COVID-19 admitted to the intensive care unit (ICU) in Chongqing, China (from January 21 to March 15, 2020).

Clinical course and outcomes		Value
**Duration from initial symptom(s) to…^a^ (days), median (IQR)**		
	Diagnosis confirmed by PCR^b^ test	5 (2-7)
	Hospital admission	7 (4-8)
	Acute respiratory distress syndrome	7 (6-10)
	ICU admission	8 (6-11)
	Ventilation	10 (7-14)
	Cessation of viral shedding^c^	20 (16-26)
	Death^d^	16 (15-28)
Length of ICU stay		13 (9-19)
Length of hospital stay		22 (16-34)
**Outcomes (n=75)**, **n (%)**		
	28-day mortality^d^	4 (5.3)
	28-day mechanical ventilation dependency	1 (1.3)
**Location of death (n=6), n (%)**		
	ICU	4 (66.7)
	Emergency department	2 (33.3)

^a^Two patients without any symptoms until hospital admission were excluded from statistical analysis.

^b^PCR: polymerase chain reaction.

^c^Two consecutive negative nasal swab PCR tests performed at an interval of least 24 hours).

^d^Four patients who died in the ICU were included.

### Early Improvement of ARDS

**Figure 1:**
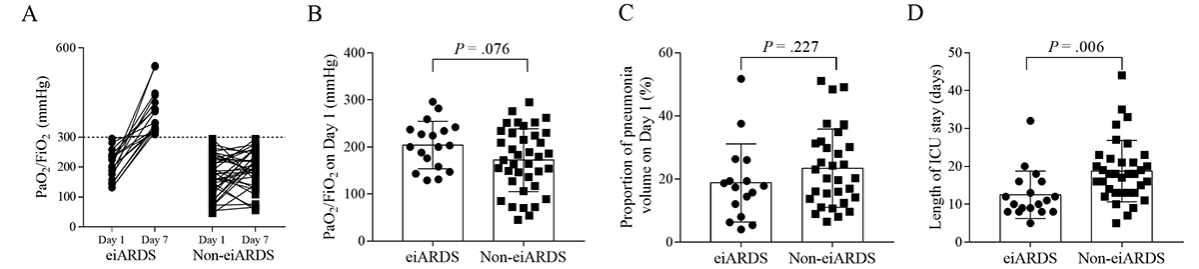
Comparison of patients with and without early improvement of acute respiratory distress syndrome (eiARDS). (A) All patients with ARDS on day 1, divided into two groups (eiARDS and non-eiARDS) according to PaO_2_/FiO_2_ on day 7. (B) No significant difference between the two groups in the PaO_2_/FiO_2_ on day 1. (C) No significant difference between the two groups in the proportion of pneumonia volume on day 1. (D) The length of intensive care unit stay exhibited differences between the two groups.

In order to clarify the characteristics of ARDS in patients with COVID-19, 56 patients with ARDS (PaO_2_/FiO_2_ <300 mm Hg) on day 1 (first day of ICU admission) were included for a subgroup analysis. These patients were then divided into two groups based on the severity of illness on day 7: eiARDS patients with PaO_2_/FiO_2_ ≥300 mm Hg and non-eiARDS patients with PaO_2_/FiO_2_ <300 mm Hg (Figure 1A).

Of the 56 patients with ARDS, a total of 18 patients had eiARDS. No significant differences were found in PaO_2_/FiO_2_ on day 1 (Figure 1B), the proportion of pneumonia volume on day 1 (Figure 1C), and the rate of ventilator usage (χ^2^=2.46, *P*=.12) between the two groups. Predictably and regrettably, all the four deceased patients did not have eiARDS. Moreover, patients with eiARDS spent a shorter duration in the ICU than those without (10.5 days, IQR 8-16 vs 18 days, IQR 13-22, respectively) (*P*=.001; Figure 1D).

A Poisson regression analysis was performed to determine the factors associated with eiARDS. Table 3 shows that two variables (age and white blood cell) with *P*<.05 in the univariate analysis were chosen for multivariable analysis. Age (<55 years) was the only variable independently associated with eiARDS, with a risk ratio of 2.67 (95% CI 1.17-6.08). This finding indicated that patients younger than 55 years were 2.67 times more likely to have eiARDS than older people.

**Table 3:** Univariate and multivariate analysis of predictors for the early improvement of acute respiratory distress syndrome (eiARDS) in patients with COVID-19 in Chongqing, China (from January 21 to March 15, 2020).

Characteristic			eiARDS (n=18)	Non-eiARDS (n=38)	Relative risk (95% CI)	*P* value
**Demographic and clinical characteristics**						
	**Age (years)^a^, n (%)**					
		≥55	6 (33.3)	26 (68.4)	1 (reference)	—^b^
		<55 (univariate)	12 (66.7)	12 (31.6)	2.67 (1.17-6.08)	.02
		<55 (multivariate)	—	—	2.36 (1.05-5.23)	.04
	Male sex (vs female), n (%)		12 (66.7)	19 (50.0)	1.61 (0.71-3.70)	.26
	Time of symptom onset to intensive care unit admission, median (days)		7.5	8	1.02 (0.92-1.14)	.69
	Smoking, n (%)		4 (22.2)	4 (10.5)	1.71 (0.75-3.90)	.20
	Hypertension, n (%)		2 (11.2)	7 (18.4)	0.65 (0.18-2.36)	.52
	Diabetes, n (%)		4 (22.2)	9 (26.3)	0.86 (0.34-2.18)	.75
	Chronic obstructive pulmonary disease, n (%)		1 (5.6)	3 (7.9)	0.77 (0.13-4.36)	.76
	APACHE II^c^, median		6.5	9	0.90 (0.76-1.05)	.17
	SOFA^d^, median		4	3	0.97 (0.67-1.42)	.89
	**Temperature, n (%)**					
		<37.3 °C	15 (83.3)	18 (47.4)	1 (reference)	—
		≥37.3 °C (univariate)	3 (16.7)	20 (52.6)	0.33 (0.11-1.02)	.054
	**Heart rate per min, n (%)**					
		<100	13 (72.2)	33 (86.8)	1 (reference)	—
		≥100	5 (27.8)	5 (13.2)	1.77 (0.82-3.83)	.15
	**Respiratory rate per min, n (%)**					
		<30	15 (83.3)	37 (97.4)	1 (reference)	—
		≥30	3 (16.7)	1 (2.6)	1.17 (0.48-2.86)	.73
	**Systolic pressure, n (%)**					
		<140 mm Hg	16 (88.9)	35 (92.1)	1 (reference)	—
		≥140 mm Hg	2 (11.1)	3 (7.9)	1.28 (0.41-4.02)	.68
		Ventilation (vs non)	2 (11.1)	5 (13.2)	0.88 (0.25-3.02)	.83
		Proportion of pneumonia volume, median	17.6	20.3	0.98 (0.94-1.02)	.28
**Laboratory findings**						
	**pH, n (%)**					
		7.35-7.45	4 (22.2)	12 (31.6)	1 (reference)	—
		>7.45	14 (77.8)	26 (65.8)	1.44 (0.56-3.70)	.45
	**PaCO_2_^e^, n (%)**					
		34-45 mm Hg	9 (50.0)	21 (55.3)	1 (reference)	—
		≤34 mm Hg	8 (44.4)	17 (44.7)	1.07 (0.48-2.35)	.87
	**PaO_2_^f^, n (%)**					
		≥60 mm Hg	14 (77.8)	23 (60.5)	1 (reference)	—
		<60 mm Hg	4 (22.2)	15 (39.5)	0.56 (0.21-1.46)	.23
	**White blood cell count (×10^9^/L)^a^, n (%)**					
		<4 (univariate)	7 (38.9)	6 (16.2)	2.21 (1.04-4.73)	.04
		<4 (multivariate)	—	—	1.94 (0.97-3.86)	.06
		4-10	9 (50.0)	28 (75.7)	1 (reference)	—
		>10 (univariate)	2 (11.1)	3 (8.1)	1.64 (0.49-5.54)	.42
		>10 (multivariate)	—	—	1.37 (0.48-3.89)	.55
	**Lymphocyte (×10^9^), median**		0.8	0.8	2.18 (0.79-6.02)	.13
	**Platelet (×10^9^/L), n (%)**					
		≥100	16 (88.9)	35 (94.6)	1 (reference)	—
		<100	2 (11.1)	2 (5.4)	1.59 (0.55-4.60)	.39
	**Potassium, n (%)**					
		3.5-4.5 mmol/L	14 (77.8)	25 (67.6)	1 (reference)	—
		<3.5 mmol/L	3 (16.7)	11 (29.7)	0.61 (0.21-1.82)	.38
	**Sodium, n (%)**					
		135-145 mmol/L	10 (55.6)	20 (54.1)	1 (reference)	—
		<135 mmol/L	8 (44.4)	16 (43.2)	1.00 (0.47-2.14)	>.99
	**Albumin, n (%)**					
		≥40 g/L	3 (17.6)	4 (10.8)	1 (reference)	—
		<40 g/L	14 (82.4)	33 (89.2)	0.70 (0.27-1.82)	.46
	**Total bilirubin, n (%)**					
		≤17.1 μmol/L	10 (55.6)	27 (71.1)	1 (reference)	—
		>17.1 μmol/L	8 (44.4)	11 (28.9)	1.56 (0.74-3.29)	.25
	**Alanine aminotransferase, n (%)**					
		≤40 U/L	13 (72.2)	23 (60.5)	1 (reference)	—
		>40 U/L	5 (27.8)	15 (39.5)	0.69 (0.29-1.66)	.41
	**Creatine kinase, n (%)**					
		≤200 U/L	13 (81.2)	28 (75.7)	1 (reference)	—
		>200 U/L	3 (18.8)	9 (24.3)	0.67 (0.27-2.32)	.79
	**High-sensitivity cardiac troponin T, n (%)**					
		≤0.014 ng/mL	9 (90.0)	19 (86.4)	1 (reference)	—
		>0.014 ng/mL	1 (10.0)	3 (13.6)	0.78 (0.13-4.62)	.78
	**D-dimer, n (%)**					
		≤0.55 μg/L	9 (81.8)	15 (39.5)	1 (reference)	—
		>0.55 μg/L	2 (18.2)	23 (60.5)	0.96 (0.40-2.33)	.93
	**Procalcitonin, n (%)**					
		≤0.046 ng/ml	8 (53.3)	6 (16.7)	1 (reference)	—
		>0.046 ng/ml	7 (46.7)	30 (83.3)	0.49 (0.23-1.05)	.07
	High-sensitivity C reaction protein (mg/L), median		44.6	95.2	0.99 (0.98-1.00)	.07

^a^Two variables (age, white blood cell count) were chosen for multivariable analysis.

^b^Not applicable.

^c^APACHE: acute physiology and chronic health evaluation.

^d^SOFA: sequential organ failure assessment.

^e^PaCO_2_: arterial partial pressure of carbon dioxide.

^f^PaO_2_: arterial partial pressure of oxygen.

## Discussion

### Principal Findings

In this study, the mortality rate associated with COVID-19 in Chongqing was 1.04%, and the 28-day case fatality rate of ICU patients was 5.3%. ARDS developed in 93% of ICU patients, and HFNC was the most commonly used type of oxygen therapy. About one-third of patients with ARDS improved in 1 week, which we defined as eiARDS. Patients younger than 55 years were more likely to exhibit eiARDS.

The mortality of COVID-19 varied widely across different periods and areas. In the early stage of the outbreak, Wuhan reported a mortality rate of 4.3% among hospitalized patients [15] and 61.5% among critically ill patients [16]. However, mortality in ICU patients gradually decreased to 32.5%-38.5% as time elapsed [17,18]. On the other hand, it was 26% in Lombardy, Italy [19], and 50% in Seattle, United States [20]. In the present study, a case fatality rate of only 5.3% was reported in ICU patients in Chongqing. The large differences in mortality were probably because medical resources could not be supplied timely, including health workers and hospital beds [21]. As a matter of fact, a substantial number of health workers from other provinces provided aid to Hubei Province, alongside an increase in acute care beds [21]. Similar to the model of Hubei Province, as the first cluster cases of COVID-19 were detected in Chongqing, four designated hospitals were arranged and prepared for only patients with COVID-19, and medical experts were invited from different hospitals in Chongqing. The centralized dispatch of medical resources was key for treating COVID-19 in China.

ARDS is the primary factor that increases mortality. According to the Berlin definition, stages of mild, moderate, and severe ARDS were associated with increased mortality (27%, 32%, and 45%, respectively) [12]. Studies showed that ARDS was one of the risk factors for death in patients with COVID-19 [16,22]. Much effort has been made to treat ARDS. However, only mechanical ventilation was shown to be effective [23]. Interestingly, in this study, although 93% of patients were affected by ARDS, only 35% received noninvasive ventilation and 9% received invasive ventilation. The most commonly used oxygen therapy was HFNC, which accounted for 55% (although patients may receive both HFNC and ventilation), meaning that HFNC was effective for COVID-19–induced ARDS. Similar conclusions were observed in a previous study [4]. The authors held that HFNC was suitable for patients with COVID-19 and mild ARDS, and even safe for patients with moderate and severe outcomes, which was clearly inconsistent with the stratified treatment strategies of ARDS caused by other factors [4].

In our study, nearly one-third of patients with ARDS recovered in 1 week, which we defined as eiARDS. However, eiARDS was found in only 18% of patients with mild ARDS caused by other factors, with 36% of patients persisting and 46% worsening during the first week after ARDS onset [24]. It is worth mentioning why so many patients with COVID-19 had eiARDS and why HFNC oxygen therapy was so effective for these patients. Gattinoni et al [25] proposed two types of patients with COVID-19 pneumonia: non-ARDS type 1 and ARDS type 2. Although both types of patients met the ARDS Berlin definition, severe hypoxemia in type 1 patients was associated with nearly normal respiratory system compliance, which led to ventilation/perfusion mismatch [25]. The aforementioned problems could be explained if we assume that patients with type 1 pneumonia would improve quickly compared with those with type 2. In addition, Gattinoni et al [25] proposed that the gas volume and percentage of nonaerated tissue could be clearly distinguished via CT scan between type 1 and type 2 pneumonia. However, no differences were found in the proportion of pneumonia volume between eiARDS and non-eiARDS patients in this study.

Regardless of the reasons why the proportion of eiARDS was high, paying attention to eiARDS itself is clinically meaningful. Early or rapid improvements in ARDS has always been associated with increased survival or better outcomes [24,26]. For patients with COVID-19, early improvement in oxygenation was associated with being discharged alive from the ICU [22]. Patients with eiARDS were found to have a higher survival rate and lower length of ICU stay compared with non-eiARDS patients. Dynamic observation of ARDS in the short term was worthwhile for the prognosis of COVID-19, and patients whose ARDS did not improve in 1 week should be given more attention. If most patients had mild ARDS at baseline, that would explain the higher percentage of patients with eiARDS. However, at baseline, 38.8% of patients had moderate ARDS, and no significant differences were found between patients with and without eiARDS. In other words, the initial PaO_2_/FiO_2_ value was independently associated with eiARDS. Indeed, multiple studies showed that older age (>65 years) was one of the risk factors for death in patients with COVID-19 [16,27-29] and establishing risk stratification through age (>60 years) might be helpful to clinicians [30]. Similar underlying mechanisms might be identified with regard to the effect of age on death and the development of ARDS. Nevertheless, age should be given immense attention during the management of patients with COVID-19.

### Limitations

This study had several limitations. First, because of the retrospective study design, laboratory tests (except arterial blood gas analysis, which was performed daily) might not be performed for all patients at a specific time; the missing data were replaced by values obtained within the prior 2 days. Second, although the treatment strategies of the two hospitals were in accordance with the guidelines issued by the Chinese National Health Commission, some of the treatments were different, such as the composition of traditional Chinese medicine, leading to different clinical outcomes. Third, the sample size was relatively small, and some of the conclusions need to be verified using multiple care centers and larger sample sizes.

### Conclusions

A new subphenotype of ARDS—eiARDS—in patients with COVID-19 was identified. As clinical outcomes differ, the stratified management of patients based on eiARDS or age is highly recommended.

## Abbreviations

ARDS: acute respiratory distress syndrome
CT: computed tomography
eiARDS: early improvement of ARDS
FiO_2_: oxygen concentration
HFNC: high-flow nasal cannula
ICU: intensive care unit
PaO_2_: arterial blood oxygen partial pressure
PCR: polymerase chain reaction
RT-PCR: real-time reverse transcription–polymerase chain reaction
ULN: upper limit of normal
WHO: World Health Organization
